# Changes in experienced quality of oncological cancer care during the COVID-19 pandemic based on patient reported outcomes – a cross-sectional study

**DOI:** 10.2340/1651-226X.2024.40141

**Published:** 2024-06-30

**Authors:** Ninna Aggerholm-Pedersen, Lise Bech Jellesmark Thorsen, Nina Møller Tauber, Josefine Tingdal Danielsen, Katrine Løppenthin, Signe Borgquist, Christoffer Johansen, Robert Zacharie

**Affiliations:** a Department of Oncology, Aarhus University Hospital, Aarhus, Denmark; b Department of Clinical Medicine, Aarhus University, Aarhus, Denmark; c Unit for Psychooncology and Health Psychology, Department of Psychology and Behavioural Science, Aarhus University, Aarhus, Denmark; d Department of Oncology, Rigshospitalet, København, Denmark; e Center for Surgery and Cancer, Rigshospitalet, University of Copenhagen, Copenhagen, Denmark; f Center Late Effect Research, Oncology Clinic, Copenhagen, Denmark

**Keywords:** COVID-19, patient-reported outcome, questionnaires, cancer

## Abstract

**Aim:**

The study aims to investigate the impact of the COVID-19 pandemic on cancer patients’ perceptions of the quality of their oncological treatment and care.

**Background:**

The COVID-19 pandemic disrupted healthcare delivery and oncological resources were repurposed, potentially leading to prolonged treatment and reduced access to innovative therapies and clinical trials. Still, little is known about how patients perceived the quality of their treatment.

**Methods:**

A cross-sectional study was conducted in the spring of 2020 among cancer patients at the Department of Oncology, Aarhus University Hospital and Rigshospitalet, Denmark. Patients were invited to complete an online questionnaire on clinical, socioeconomic, emotional, behavioural, and quality-related aspects of oncological cancer care. Patients who experienced reduced treatment quality and those who reported no or slight reductions were compared using multiple logistic regression, exploring the associations with patient characteristics, behaviours, and fear of cancer progression or recurrence.

**Results:**

A total of 2,040/5,372 patients experienced changes in their treatment plans during the pandemic, and 1,570/5,372 patients experienced reduced treatment quality, with 236 reporting a high degree of reduction. Patients with breast, head and neck, and upper gastrointestinal cancers were more likely to experience reduced treatment quality. Altered interactions with healthcare providers, along with isolation, lack of social support, and heightened fear of cancer progression, were significant risk factors for experiencing reduced cancer care quality.

**Interpretation:**

We identified subgroups of cancer patients needing targeted communication and care during health crises affecting cancer treatment. The findings underscore the importance of safeguarding the needs of vulnerable patient populations in future healthcare emergencies.

## Introduction

The COVID-19 pandemic exposed cancer patients to exceptional vulnerabilities due to the inherent life-threatening nature of their condition. These vulnerabilities stemmed from a convergence of factors, including pandemic-induced limitations on hospital visits, apprehensions about COVID-19 contagion, and concerns regarding timely detection and intervention for cancer progression or recurrence [1].

In response to the pandemic, the European Organization for Research and Treatment of Cancer (EORTC) recommended changing oncological routines and suggested contingency plans for modifying cancer care if forced by the circumstances [2–7]. Such changes could include temporarily deferring cancer screening, delaying or avoiding outpatient visits, and postponing elective surgery and systemic cancer treatment in patients [6, 8–11].

Such changes in cancer care can place an additional burden on patients, increasing the substantial toll of cancer diagnosis and treatment on psychological well-being and physical health [12, 13]. A recent meta-analysis, including 27,590 cancer patients during the COVID-19 pandemic, indicated that approximately one-third of the participants suffered from clinical levels of depression and anxiety and that almost two-thirds reported heightened fear of cancer progression or recurrence [14].

To the best of our knowledge, no previous studies have explored how cancer patients experienced the changes in their cancer care during the pandemic. In this study, we examined data from a tax-funded, universal accessible healthcare system in the two most populated regions of Denmark. Specifically, we present patient-reported outcome (PRO) data on the changes experienced by patients during the pandemic in the quality of treatment, care, and follow-up, exploring the associations between experienced reductions in the quality of their treatment, fear of SARS-CoV-2 infection, and fear of cancer progression or recurrence.

## Patients and methods

### Patient selection

Our survey study enrolled patients in active treatment or follow-up care at the two largest oncology departments in Denmark: The Department of Oncology, Aarhus University Hospital (AUH), and the Department of Oncology, Rigshospitalet (RH). Collectively, the two departments provide oncological care to more than 1/3 of all Danish residents. The Danish healthcare system is based on the principle of universal health coverage, and all Danish residents, irrespective of their socio-economic status, have equal access to healthcare at all levels, from general practitioners to highly specialised hospital departments, including cancer treatment.

Patients were invited to participate in the study via a secure national electronic mail system linked to the Danish civil registration number, a unique personal 10-digit identifier assigned to all Danish residents since 1st April 1968. More than 90% of Danish adults have access to a secure electronic mail account, routinely utilised by public administration, including healthcare providers, for communication. At AUH/RH, study enrolment lasted from March 11, 2020 (the official lockdown date in Denmark) to May 27, 2020. Patients consenting to participate received a link to an electronic questionnaire. REDCap [15], a GDPR-compliant electronic data capture platform administered by the Aarhus University Clinical Trial Unit, was used for data acquisition. If patients did not answer the questionnaire, a reminder was sent.

### Patient-reported outcomes

Participants were asked to complete a comprehensive questionnaire including between 107 and 122 items, depending on the responses provided, with an estimated completion time of 20–30 minutes after giving written consent to participate; no exclusion criteria were present in this study. The instruments and references to the questionnaires are listed in Appendix 1. In brief, the questionnaire included scales and individual items covering the following domains: (1) demographic information, for example, marital status, children living at home; (2) clinical details, including cancer diagnosis and type of treatment; (3) questions on any previous or suspected COVID-19 infections; (4) *ad hoc* questions on perceived changes in cancer treatment and care; (5) a six-point scale assessing physical activity together with single items on health behaviors, for example, smoking, nutrition, and alcohol. The patients were also asked to rate (6) physical health, (7) social distancing behaviors, (8) fear of SARS-CoV-2 infection, and (9) fear of cancer progression or recurrence, including a question about whether the current COVID-19 situation had worsened their fear of cancer progression or recurrence. Additional topics included (10) perceived stress, (11) sleep duration, sleep disturbance, and sleep quality, (12) social support and social isolation, (13) items on depression, anxiety, fatigue, and general quality of life (QoL), and, finally, (14) an open-ended question, allowing patients to report any additional aspects they found relevant.

### Data analysis

To verify the cancer diagnosis for the responder and non-responder groups, we linked the self-reported data to clinical data from the patient’s medical records using the hospital electronic database, specifically for patients treated at AUH. Differences between responders and non-responders were analysed using *t*-tests or Chi^2^ tests, as appropriate for the respective data types. Due to data privacy concerns, RH did not authorise access to data on non-responders, precluding a responder–non-responder analysis for this center.

In the present report, we examine the patient perspective concerning treatment quality and focus on its correlations with two key factors: fear of SARS-CoV-2 infection and fear of cancer progression or recurrence. Fear of SARS-CoV-2 infection was assessed with a seven-item scale [16], with each item rated on a scale from 1 to 5. The total score ranged from 5 to 35, with higher scores indicating higher levels of fear. Based on the suggested cut-off of 16 [16], patients were dichotomised into a low (≤ 16) and high level of fear group (> 16). Fear of cancer recurrence or progression was measured with the 3-item version of the Concerns About Recurrence Questionnaire (CARQ-3) [17], with three 11-point (0–10) numerical rating scales yielding a total score from 0 to 30. Using the suggested cut-off, patients were categorised as either expressing minimal concern about their disease (≤ 10) or experiencing heightened worry (> 10).

The remaining independent variables were analysed as continuous variables and included depression [18], emotional, informational, and instrumental social support [19], and social isolation [19]. The questionnaire details, including internal consistencies (Cronbach’s alpha) calculated for the answers provided in this study, are shown in Table 1.

**Table 1:** Internal consistency in the different scores used. The results are based on the patients’ responses in this study.

Questionairs	Number of items	Cronbach’s alfa	Mean	Sd	*n*
Emotional [19]	4	0.92	17.0	4.5	5,484
Informative [19]	4	0.90	15.7	4.6	5,484
Instrumental [19]	4	0.93	16.9	4.9	5,484
Social [19]	4	0.80	7.0	3.5	5,484
Physical Function [19]	6	0.91	24.3	7.7	5,484
PSS (stress) [20]	4	0.66	4.9	3.1	5,484
Depression [18]	2	0.83	4.4	4.9	5,484
Anxiety	2	0.86	4.1	4.9	5,484
Tiredness	2	0.85	6.9	5.6	5,484
Pain	2	0.85	5.0	5.7	5,484
Fear of SARS-CoV-2 infection [21]	7	0.88	15	5.7	5,376
Fear of cancer recurrence [17]	3	0.92	6.6	8.4	5,484
Fear of cancer progression	3	0.93	4.0	7.9	5,484

The primary endpoint was patient-reported perceived change in treatment quality. The investigated predictors of perceived reductions in treatment quality were analysed with a hierarchical logistic regression analysis. The analysis involved six consecutive steps. Variables at each step reaching statistical significance (*p* < 0.05) were carried forward and adjusted for at the next step, advancing from the more distal demographic background factors (step 1) over the clinical characteristics (step 2), to health behaviors and physical function (step 3), psychological and physical symptoms (step 4), and aspects of social support (step 5). Finally (step 6), all variables reaching statistical significance at the 5% level at the fifth step were entered together in a final model. All statistical analyses were performed using STATA 17.0 (StataCorp LLC, USA).

### Ethics and data protection

The study was approved by the Danish Patient Safety Authority (Record no., 31-1521-376) and the Danish Data Protection Agency (Record no., 1-16-02-143-20).

## Results

**Figure 1:**
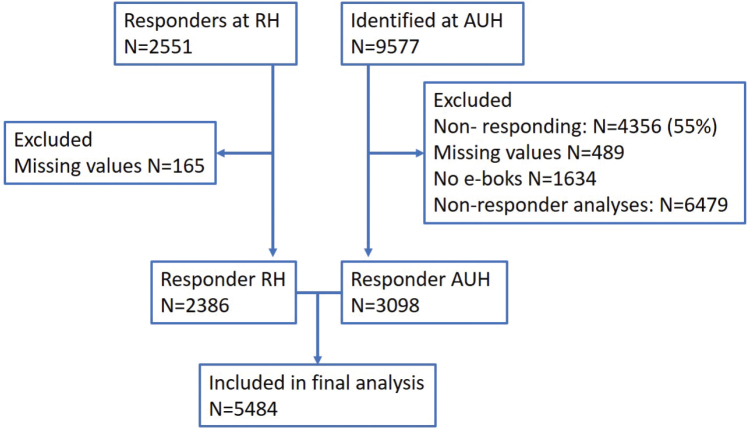
The inclusion of patients for Aarhus University Hospital (AUH) and Rigshospitalet (RH). Missing values are patients excluded due to missing information in essential variables in the questionnaires.

At AUH, a total of 3,587 patients responded to the questionnaire, corresponding to a response rate of 3,587/7,943 (45%). An additional 2,386 patients from RH responded. Participant recruitment and flow is illustrated in Figure 1. A responder–non-responder analysis for patients at AUH is summarised in Table 2. Comparing characteristics of patients from AUH and RH showed that a higher percentage of the patients at RH were single or separated (marital status), had an intermediate or long higher education, had children living at home, and had a full-time position at work. The distributions of treatment modality and treatment intent between the centres were comparable (Table 3).

**Table 2:** Comparing responders and non-responders treated at Aarhus University Hospital (AUH).

Variables	Responders	%	Non-responders	%	*p*
Total number	3,098		6,479		
Gender					
Women	1,663	54	3,599	56	
Men	1,435	46	2,880	44	0.086
Age					
Median age (5–95 perc.)	67(42–80)		68(36–84)		<0.001
Age categories					
≤20	<5	<1	18	0	<0.001
21–30	33	1	167	3	
31–40	86	3	254	4	
41–50	229	7	520	8	
51–60	589	19	1,043	16	
61–70	1,043	34	1,653	26	
71–80	1,001	32	2,085	32	
81–90	112	4	693	11	
≥ 90	<5	<1	46	1	
Diagnosis					
Breast	837	27	1,479	23	<0.001
Urogenital	799	26	1,444	22	
Lung	486	16	1,092	17	
Upper gastrointestinal	123	4	327	5	
Bowel	154	5	329	5	
Head and neck	137	4	327	5	
Sarcoma	195	6	294	5	
Melanoma	126	4	156	17	
Other	241	8	880	14	

**Table 3:** Comparison of the two cohorts from Aarhus University Hospital and Rigshospitalet.

Variables	Total	AUH	%	RH	%	*p*
**Patient characteristics**	5,484	3,098		2,386		
Gender						
Women	3,277	1,663	54	1,614	68	
Men	2,207	1,435	46	772	32	<0.001
Age						
Median (5–95 percentile), years	65 (40–79)	67 (42–80)		62 (38–77)		<0.001
Marital status						
Married	3,899	2,361	76	1,538	64	
Single/divorced/widowed	1,576	731	24	845	35	<0.001
Education level						
Primary school/high school	953	656	21	297	12	
Short/intermediate higher education	3,212	1,829	59	1,383	58	
Long higher education	954	370	12	584	24	
Other not defined or missing	365	243	8	122	5	<0.001
Working status						
Stable work	1,913	915	30	998	42	
Retirement	2,829	1,818	59	1,011	42	
No work/temporary worker	493	229	7	264	11	
Other or missing	249	136	4	113	5	<0.001
Children						
Living at home	974	481	16	493	21	
Not living at home	3,459	2,097	68	1,362	57	
None	1,047	516	17	531	22	<0.001
**Disease-related factors**						
Cancer diagnosis						
Breast	1,793	837	27	956	40	
Urogenital	1,277	799	26	478	20	
Lung	993	486	16	207	9	
Upper gastrointestinal	295	123	4	171	7	
Bowel	274	154	5	120	5	
Head and neck	254	137	4	117	5	
Sarcoma	211	195	6	16	1	
Melanoma	153	126	4	27	1	
Other	535	241	8	294	12	<0.001

AUH: Aarhus University Hospital; RH: Rigshospitalet.

**Figure 2:**
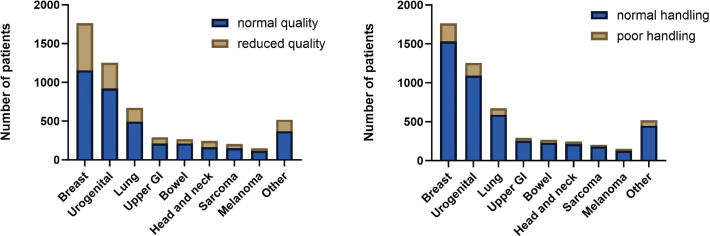
(A) Patients experiencing unchanged or reduced quality of their treatment according to cancer diagnosis. (B) The number of patients in each diagnostic category who felt that the Department of Oncology responded poorly to the COVID-19 crisis.

At the onset of the pandemic, 38% of patients experienced changes in treatment or follow-up program. Of these, a majority (56%) indicated a perceived reduction in treatment quality. Among patients who did not report changes in treatment, 421 still perceived a decline in treatment quality, yielding a total of 1,569 out of 5,372 (29%) patients who perceived the quality of their treatment to be reduced. Of these, 236/1,569 (15%) experienced an exceptionally high degree of reduction in the quality of treatment and care (See Figure 2).

**Figure 3:**
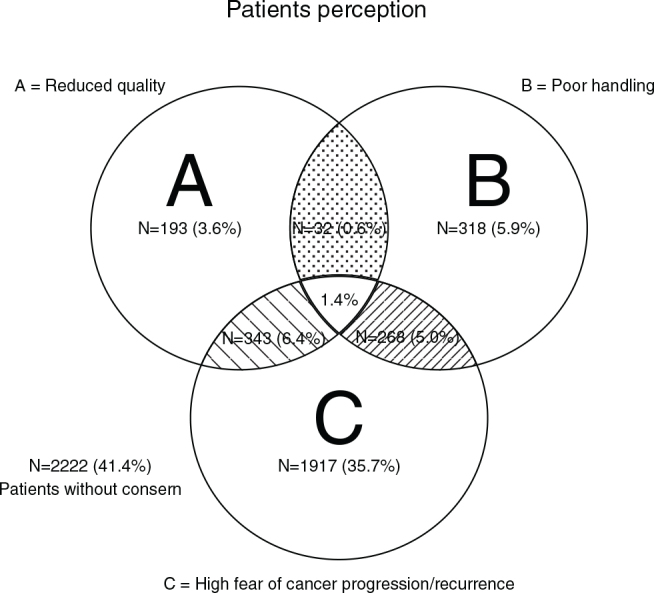
Patient perception of reduced quality, poor pandemic response by the Department of Oncology, and the proportion of patients who experienced changes in their treatment.

Concerns regarding the management of pandemic related challenges by the Department of Oncology were also raised, with 13% of the patients expressing dissatisfaction in this regard. Among those dissatisfied patients, 28% perceived a decline in treatment quality. Figure 3 illustrates a Venn diagram depicting the overlap between patients’ perceptions of reduced treatment quality, the department’s pandemic response, and alterations in their treatment plans.

Patients who found that the department did not handle the situation well, were more likely to experience a reduced quality in their treatment (OR = 1.44; 95% CI: 1.15–1.80; *p* < 0.001). As seen in Table 4, experiencing a reduced treatment quality was associated with experienced changes in contact with the department or other health care providers. The available data did not enable us to determine whether the visits were unscheduled or scheduled.

**Table 4:** Results of univariate logistic regression exploring the associations of changes in treatment and contacts with the oncology department with perceived reduced treatment quality.

Questions	Total	Reduced Quality		
		OR	95%CI	*p*
**Changes in cancer treatment/Contact**				
Contact to the Department (vs. unchanged)	4,890			
Less Contact	335	4.09	3.19–5.24	<0.001
More Contact	152	3.25	2.24–4.70	<0.001
Visit at the Department (vs. unchanged)	4,729			
Fewer visits	623	3.18	2.59–3.89	<0.001
More visits	22	5.11	2.13–12.24	<0.001
Contact to GP (vs. unchanged)	4,469			
Fewer contacts	775	2.23	1.88–2.79	<0.001
More contacts	128	2.46	1.60–3.78	<0.001
Admitted to the hospital since March 11, 2020 (vs not)	588	1.60	1.27–2.02	<0.001

Higher scores on emotional, informative, or instrumental support correlated with greater treatment satisfaction. Conversely, patients reporting higher levels of depression, anxiety, pain, and fatigue were more likely to report reduced treatment quality and heightened fears of cancer progression or SARS-CoV-2 infection.

Lack of trust in the handling of the pandemic by Department of Oncology increased the perception of reduced treatment quality. The final adjusted logistic regression model showed that female gender, younger age, active treatment, social isolation, pain, and fear of cancer recurrence/progression were all associated with reduced perceived quality of treatment (Table 5).

**Table 5:** Logistic regression results comparing different independent variables to patients who found reduced treatment quality.

Independent variables	*N*	Unadjusted analyses			Adjusted analysis*		
		OR	95%CI	*p*	OR	95%CI	*p*
**Final model**							
Sex (ref: female)	5,484	-	-	-	1.29	1.01–1.64	0.038
Age	5,484	-	-	-	0.99	0.98–1.00	0.017
Active Treatment (ref: no or hormone treatment)	5,417	-	-	-	1.60	1.16–2.21	0.004
Active curative/palliative treatment(ref: follow-up program)	5,432	-	-	-	1.54	1.11–2.14	0.009
Social isolation	5,371	-	-	-	1.06	1.03–1.10	<0.001
Pain	5,371	-	-	-	1.04	1.02–1.06	<0.001
Fear of cancer recurrence/progression	5,371	-	-	-	1.04	1.02–1.05	<0.001
	-	-	-	-	-	*R*^2^ = 0.059	

Significant results used in the hierarchical multivariate analysis are shown in Supplementary Table 1. For the different scoring systems, a higher score indicated higher odds of patients reporting reduced quality of the treatment.

* Significant results used in the hierachical multivariat analysis. The significant levels are 0.05

A total of 2,259/5,371 (42%) patients reported fear of being infected by SARS-CoV-2. There was no association between patients’ fear of being infected by SARS-CoV-2 and their satisfaction with the department’s pandemic response. Patients with high levels of fear of SARS-CoV-2 infection were also more likely to experience higher levels of fear of cancer progression or recurrence (1,466/2,259; 65%). A higher percentage of patients with COVID-19 symptoms were worried about cancer progression or recurrence than patients without COVID-19 symptoms (56% vs 47%, respectively).

## Discussion

The results from our survey, to which all cancer patients at the two largest oncological treatment centres in Denmark were invited to participate, indicate that the COVID-19 pandemic affected the cancer patients’ experience of their treatment, with one-third of patients reporting changes in their treatment or follow-up program. Female sex, younger age, undergoing ,active cancer treatment, being socially isolated, being in pain, and experiencing high levels of fear of cancer progression or recurrence, were all factors that increased the probability of reporting reduced oncological treatment quality during the COVID-19 pandemic. While patients experiencing high levels of fear of SARS-CoV-2 infection also experienced reductions in the quality of their treatment, they were not more dissatisfied with how the oncology department had handled the pandemic.

Our study provides a unique insight into the well-being of cancer patients during a pandemic. Particular strengths are the non-responder versus responder analyses and the comparison between RH and AUH, with RH serving as the capital of Denmark and AUH serving as not only the second-largest city but also smaller towns and villages.

Our finding that more than one-third of the patients had experienced changes in their treatment or follow-up program at the start of the pandemic is consistent with results of other studies showing that 25–30% of patients reported such changes [22–24]. A British study [25] found that the pandemic significantly impacted radiation therapy, increasing the use of hypofractionated radiation therapy, but whether such changes may have influenced the outcomes of interest in our study is unclear. The contact with the health care providers changed for 9.1% of the patients in our study, a percentage similar to that found in a smaller study (*n* = 366) of melanoma patients, in which 10.1% reported changes in their appointments due to the pandemic [26]. Our results show that an altered frequency, both higher or lower, of contacts with either the Department of Oncology or the GP was associated with perception of reduced treatment quality. We found no associations between perceived reduced treatment quality, perceptions of how the oncology departments handled the pandemic, and the frequency of contacts with the healthcare system. This appears to indicate that perception of reduced treatment quality is associated with change in general, rather than changes in a specific direction.

A meta-analysis of 40 studies conducted during the COVID-19 pandemic revealed that among cancer patients roughly 33% reported depression, 31% anxiety, and 67% fear cancer progression or recurrence. Our study observed lower rates of these outcomes, with approximately 11% showing signs of depression or anxiety and 47% expressing a fear of progression or recurrence. The difference between the results from the meta-analysis and our results could be explained by several factors. One explanation could be differences in the measures used. Another could be the overrepresentation of breast cancer patients in our study and the inclusion of cancer survivors in follow-up programs, who may experience fewer symptoms of depression and anxiety compared with those undergoing active treatment for their disease.

Fear of cancer progression and recurrence has consistently been shown to be associated with higher levels of psychological distress and impaired quality of life [27, 28]. Our results indicated that patients with breast cancer and a high fear of cancer progression or recurrence were more likely to perceive poorer treatment quality, suggesting that changes to treatment and appointment schedules might be perceived as particularly threatening by some patient groups. Furthermore, patients who missed social interaction with others during the pandemic and who felt socially isolated also reported reduced treatment quality. This is consistent with previous studies showing that social isolation and loneliness can negatively impact cancer patients’ quality of life and psychological well-being [29]. Providing appropriate support for cancer patients during the pandemic is essential to address their fears and concerns.

Half the patients report being afraid of being infected by SARS-CoV-2; these patients were primarily women, lung cancer patients, those living alone, and patients undergoing curative-intended treatment. Lung cancer patients may have higher anxiety due to their perceived susceptibility to severe infection. Similarly, patients living alone may experience increased fear due to limited social support, while those undergoing curative-intended treatment may worry about the potential impact of infection on their treatment outcomes. The percentage of patients with cancer being afraid of SARS-CoV-2 infection is similar to the percentage of the general population. A German survey reported that 59% of participants were afraid of being infected with SARS-CoV-2 [30].

The generalizability of our results might be questioned. The response rate among patients from AUH was as anticipated but relatively low at approximately 45%. The limited response, along with the variation between responders and non-responders, was elucidated though responder–non-responder analysis. Additionally, the absence of data regarding the number of patients invited at RH reduces the study’s interpretive scope. Another limitation is the potential selection bias, for example, related to socio-economic status (SES) [31]. However, patients in Denmark have equal access to medical care in the public health care system; therefore, selection bias caused by socio-economic factors should not play a major role in the study results. Furthermore, cancer treatment in Denmark is guided by national guidelines that aim to ensure uniform, high-quality, evidence-based care for all patients. This does not exclude the possibility of selection bias due to excluding patients with missing data on crucial variables and patients who could not receive the questionnaire through the public electronic communication tool. A second potential limitation could be the relatively large number of variables included in the analyses. We have attempted to balance the risk of over- and underfitting the data by conducting a hierarchical multiple logistic regression analysis, selecting only statistically significant variables to be carried forward to the next step but choosing a reasonably liberal significance level (5%). Still, the regression analysis results should be interpreted with some caution, as some degree of multicollinearity between the variables does exist. Third, the study relied on self-reported data, which could have introduced misclassification insofar that patients might be unclear on their specific diagnosis, and some may be unaware of the aim of their treatment. Finally, although our findings generally appear to be consistent with the results of previous studies, some results may not be generalisable to other healthcare systems. In our study, we found that separating patients undergoing treatment from those in follow-up or treatment pause is not straightforward. This is mainly because distinguishing between patients in treatment pause with residual disease and those without any evidence of disease during follow-up is impossible. Additionally, patients in active treatment included both palliative patients and those receiving adjuvant treatment, who may have markedly different clinical profiles; therefore, this stratification was not made.

## Conclusion

In conclusion, our study highlights the fact that many patients experienced changes in treatment during the outbreak of the COVID-19 pandemic, with reduced quality in cancer treatment, particularly among patients with specific cancer types. Dissatisfaction with how the Department of Oncology handled the pandemic challenges was linked to the perception of reduced treatment quality. Additionally, the study showed how social support in patient satisfaction is essential, and the impact of psychological factors such as depression and anxiety are linked to the treatment experiences. The study reveals the COVID-19 pandemic’s significant impact on cancer patient’s treatment experiences, highlighting the need for proactive health services planning. Strategies should focus on effective communication, addressing psychological well-being, and promoting social support by prioritising patient-centred care, even during crises.

## Author contribution

Conceptualisation, L.J.T., N.M.T., J.T.D., K.L., S.B., C.J., R.Z., and NAP; methodology, R.Z., L.J.T, and N.A.P.; formal analysis, R.Z., L.J.T., and N.A.P.; investigation, L.J.T., N.M.T., J.T.D., K.L., S.B., C.J., R.Z., and N.A.P.; data curation, L.J.T., N.M.T., J.T.D., K.L., S.B., C.J., R.Z., and N.A.P.; writing—original draft preparation, L.J.T. and N.A.P.; writing—review and editing, L.J.T., N.M.T., J.T.D., K.L., S.B., C.J., R.Z., N.A.P.; visualisation, L.J.T. and N.A.P.. All authors have read and agreed to the published version of the manuscript.

## Supplementary Material

Changes in experienced quality of oncological cancer care during the COVID-19 pandemic based on patient reported outcomes – a cross-sectional study

## Data Availability

The data generated and analysed in this study are not publicly available. This is by the rules concerning processing personal data described in the EU General Data Protection Regulation (GDPR) and the Danish Data Protection Act. However, should a researcher be interested in our data, they are welcome to contact the corresponding author, Ninna Aggerholm-Pedersen.

## References

[1] Liang W, Guan W, Chen R, et al. Cancer patients in SARS-CoV-2 infection: a nationwide analysis in China. Lancet Oncol. 2020;21(3):335–7. 10.1016/S1470-2045(20)30096-6 32066541 PMC7159000

[2] Akladios C, Azais H, Ballester M, et al. Recommendations for the surgical management of gynecological cancers during the COVID-19 pandemic – FRANCOGYN group for the CNGOF. J Gynecol Obstet Hum Reprod. 2020;49(6):101729. 10.1016/j.jogoh.2020.101729 32247066 PMC7118621

[3] Ansarin M. Surgical management of head and neck tumours during the SARS-CoV (COVID-19) pandemic. Acta Otorhinolaryngol Ital. 2020;40(2):87–9. 10.14639/0392-100X-N0783 32271745 PMC7256907

[4] Bartlett DL, Howe JR, Chang G, et al. Management of cancer surgery cases during the COVID-19 pandemic: considerations. Ann Surg Oncol. 2020;27(6):1717–20. 10.1245/s10434-020-08461-2 32270420 PMC7141488

[5] Burki TK. Cancer guidelines during the COVID-19 pandemic. Lancet Oncol. 2020;21(5):629–30. 10.1016/S1470-2045(20)30217-5 32247319 PMC7270910

[6] Catanese S, Pentheroudakis G, Douillard JY, et al. ESMO management and treatment adapted recommendations in the COVID-19 era: pancreatic cancer. ESMO Open. 2020;5(Suppl 3):e000804. 10.1136/esmoopen-2020-000804 32423899 PMC7239531

[7] de Azambuja E, Trapani D, Loibl S, et al. ESMO management and treatment adapted recommendations in the COVID-19 era: breast cancer. ESMO Open. 2020;5(Suppl 3):e000793. 10.1136/esmoopen-2020-000793 32439716 PMC7295852

[8] Di Fiore F, Bouché O, Lepage C, et al. COVID-19 epidemic: proposed alternatives in the management of digestive cancers: a French intergroup clinical point of view (SNFGE, FFCD, GERCOR, UNICANCER, SFCD, SFED, SFRO, SFR). Dig Liver Dis. 2020;52(6):597–603. 10.1016/j.dld.2020.03.031 32418773 PMC7255323

[9] Perrone AM, De Palma A, De Iaco P. COVID-19 global pandemic: options for management of gynecologic cancers. The experience in surgical management of ovarian cancer in the second highest affected Italian region. Int J Gynecol Cancer. 2020;30(6):902. 10.1136/ijgc-2020-001489 32381511

[10] The Lancet Oncology. COVID-19: global consequences for oncology. Lancet Oncol. 2020;21(4):467. 10.1016/S1470-2045(20)30175-3 32240603 PMC7118606

[11] Vecchione L, Stintzing S, Pentheroudakis G, et al. ESMO management and treatment adapted recommendations in the COVID-19 era: colorectal cancer. ESMO Open. 2020;5(Suppl 3):e000826. 10.1136/esmoopen-2020-000826 32457036 PMC7276236

[12] Chew QH, Wei KC, Vasoo S, et al. Narrative synthesis of psychological and coping responses towards emerging infectious disease outbreaks in the general population: practical considerations for the COVID-19 pandemic. Singapore Med J. 2020;61(7):350–6. 10.11622/smedj.2020046 32241071 PMC7926608

[13] Horesh D, Brown AD. Traumatic stress in the age of COVID-19: a call to close critical gaps and adapt to new realities. Psychol Trauma. 2020;12(4):331–5. 10.1037/tra0000592 32271070

[14] Zhang L, Liu X, Tong F, et al. The prevalence of psychological disorders among cancer patients during the COVID-19 pandemic: a meta-analysis. Psychooncology. 2022;31(11):1972–87. 10.1002/pon.6012 35950545 PMC9538248

[15] Harris PA, Taylor R, Thielke R, et al. Research electronic data capture (REDCap) – a metadata-driven methodology and workflow process for providing translational research informatics support. J Biomed Inform. 2009;42(2):377–81. 10.1016/j.jbi.2008.08.010 18929686 PMC2700030

[16] Lee JJ, Choi HR, Choi EP, et al. Psychometric evaluation of Korean version of COVID-19 fear scale (K-FS-8): a population based cross-sectional study. PLoS One. 2023;18(3):e0282589. 10.1371/journal.pone.0282589 36893101 PMC9997981

[17] Thewes B, Zachariae R, Christensen S, et al. The concerns about recurrence questionnaire: validation of a brief measure of fear of cancer recurrence amongst Danish and Australian breast cancer survivors. J Cancer Surviv. 2015;9(1):68–79. 10.1007/s11764-014-0383-1 25135205

[18] Aggerholm-Pedersen N, Jespersen TW, Olsen PR, et al. Pain audit as a tool to optimize pain treatment in hospital departments. Ugeskr Laeger. 2013;175(21):1491–5. 23697567

[19] Cook KF, Jensen SE, Schalet BD, et al. PROMIS measures of pain, fatigue, negative affect, physical function, and social function demonstrated clinical validity across a range of chronic conditions. J Clin Epidemiol. 2016;73:89–102. 10.1016/j.jclinepi.2015.08.038 26952842 PMC5131708

[20] Cohen S, Kamarck T, Mermelstein R. A global measure of perceived stress. J Health Soc Behav. 1983;24:385–96. 10.2307/2136404 6668417

[21] Ahorsu DK, Lin CY, Imani V, et al. The fear of COVID-19 scale: development and initial validation. Int J Ment Health Addict. 2022;20(3):1537–45. 10.1007/s11469-020-00270-8 32226353 PMC7100496

[22] Lang JJ, Narendrula A, Iyer S, et al. Patient-reported disruptions to cancer care during the COVID-19 pandemic: a national cross-sectional study. Cancer Med. 2023;12(4):4773–85. 10.1002/cam4.5270 36207994 PMC9874402

[23] Dalby M, Ailawadi N. The experience of cancer patients during the COVID-19 pandemic. J Oncol Pharm Pract. 2023;29(2):283–9. 10.1177/10781552211066891 34904465 PMC9899693

[24] de Joode K, Dumoulin DW, Engelen V, et al. Impact of the coronavirus disease 2019 pandemic on cancer treatment: the patients’ perspective. Eur J Cancer. 2020;136:132–9. 10.1016/j.ejca.2020.06.019 32683273 PMC7334940

[25] Spencer K, Jones CM, Girdler R, et al. The impact of the COVID-19 pandemic on radiotherapy services in England, UK: a population-based study. Lancet Oncol. 2021;22(3):309–20. 10.1016/S1470-2045(20)30743-9 33493433 PMC7825861

[26] Micek A, Diehl K, Teuscher M, et al. Melanoma care during one year pandemic in Berlin: decreasing appointment cancellations despite increasing COVID-19 concern. J Dtsch Dermatol Ges. 2022;20(7): 962–78. 10.1111/ddg.14799 PMC9348098 35665996

[27] Veeraiah S, Chidambaram S, Sudhakar R, et al. Psychosocial issues and concerns of cancer patients due to COVID-19 pandemic lockdown. PLOS Glob Public Health. 2022;2(9):e0000996. 10.1371/journal.pgph.0000996 36962598 PMC10021520

[28] Ripamonti CI, Massa G, Insolvibile D, et al. Fears, beliefs, and quality of life of patients with cancer vs the general population during the coronavirus disease 2019 (COVID-19) pandemic in Lombardy. Tumori. 2022;108(5):431–8. 10.1177/03008916211022848 34176373

[29] Hossain MM, Sultana A, Purohit N. Mental health outcomes of quarantine and isolation for infection prevention: a systematic umbrella review of the global evidence. Epidemiol Health. 2020;42:e2020038. 10.4178/epih.e2020038 32512661 PMC7644933

[30] Bäuerle A, Teufel M, Musche V, et al. Increased generalized anxiety, depression and distress during the COVID-19 pandemic: a cross-sectional study in Germany. J Public Health (Oxf). 2020;42(4):672–8. 10.1093/pubmed/fdaa106 32657323 PMC7454766

[31] Lu J, Wang F, Wang X, et al. Inequalities in the health survey using validation question to filter insufficient effort responding: reducing overestimated effects or creating selection bias? Int J Equity Health. 2019;18(1):131. 10.1186/s12939-019-1030-2 31438952 PMC6704697

