# Supplementary material for: Changes in experienced quality of oncological cancer care during the COVID-19 pandemic based on patient reported outcomes – a cross-sectional study
Source: Acta Oncol. 2024 Jun 30;63:40141. doi: 10.2340/1651-226X.2024.40141 (PMC11332494; doi:10.2340/1651-226X.2024.40141)
Supplement: Changes in experienced quality of oncological cancer care during the COVID-19 pandemic based on patient reported outcomes – a cross-sectional study [file AO-63-40141-s1.pdf]

Supplementary material has been published as submitted. It has not been copyedited, or typeset by Acta Oncologica

Supplementary table 1

		Unadjusted analyses			Adjusted analysis*		
**Independent variables**	N	OR	95%CI	p-value	OR	95%CI	p-value
**Step 1: Demographic characteristics**							
Gender (ref: women)							
Men	**2207**	**0.68**	**0.57-0.81**	**<0.001**	**1.34**	**1.11-1.61**	**0.002**
Age	**5172**	**0.98**	**0.98-0.99**	**<0.001**	**0.99**	**0.98-1.00**	**0.033**
Marital status (ref: single)	503						
Married	3899	0.87	0.64-1.10	0.205	0.95	0.70-1.30	0.765
Widowed	410	0.69	0.46-1.06	0.091	0.88	0.56-1.40	0.592
Divorced	663	1.03	0.73-1.44	0.880	1.23	0.77-1.64	0.535
Education level (ref: primary/high school)	953						
Short/intermediate education	3211	1.31	1.03-1.67	0.029	1.28	0.99-1.65	0.062
Long higher education	**954**	**1.51**	**1.14-2.01**	**0.004**	**1.41**	**1.04-1.91**	**0.026**
Other, not defined or missing	366	1.07	0.71-1.62	0.748	1.21	0.79-1.84	0.374
Working status (ref: employed)	1913						
Retired	**2829**	**0.70**	**0.59-0.84**	**<0.001**	0.97	0.75-1.24	0.802
Unemployed/temporary employment	493	0.94	0.70-1.26	0.666	0.89	0.65-1.20	0.438
Other or missing	249	1.08	0.74-1.58	0.698	1.19	0.79-1.78	0.410
Children(ref: children living at home)	974						
Children not living at home	**3458**	**0.66**	**0.54-0.80**	**<0.001**	0.87	0.69-1.12	0.273
No children	1048	0.71	0.55-0.92	0.010	0.81	0.61-1.08	0.149
						R^2 =	0.0129
**Step 2: clinical characteristics**							
Diagnosis (ref: urogenital cancers)	1277						
Breast	**1794**	**1.60**	**1.28-2.01**	**<0.001**	1.33	1.00-1.78	0.054
Mmelanoma	153	0.87	0.48-1.58	0.638	1.00	0.54-1.85	0.988
Lung	693	1.23	0.91-1.66	0.181	1.35	0.98-1.87	0.066
Upper gastrointestinal	294	1.62	1.12-2.35	0.010	**1.85**	**1.24-2.77**	**0.003**
Bowel	274	0.78	0.48-1.27	0.326	0.83	0.50-1.38	0.474
Head and neck	254	1.49	0.99-2.23	0.056	**1.64**	**1.08-2.49**	**0.020**
Sarcoma	211	0.93	0.56-1.55	0.793	0.95	0.57-1.59	0.852
Other	534	1.04	0.74-1.47	0.806	1.05	0.74-1.49	0.788
Treatment intent (ref: adjuvant treatment)	1597						
Curative treatment	916	1.03	0.81-1.32	0.793	1.32	0.99-1.74	0.056
Palliative treatment	649	0.96	0.72-1.26	0.750	1.32	0.97-1.79	0.077
Follow-up program	2270	0.81	0.67-0.99	0.042	**0.61**	**0.42-0.88**	**0.008**
Treatment (ref: no treatment)	2554						
Chemotherapy	630	0.95	0.72-1.26	0.741	**0.47**	**0.31-0.73**	**0.001**
Radiation therapy	380	1.06	0.76-1.49	0.719	**0.60**	**0.37-0.95**	**0.031**
Immune therapy	281	0.77	0.50-1.18	0.232	**0.46**	**0.27-0.80**	**0.006**
Hormone therapy	576	1.32	1.01-1.71	0.040	0.69	0.45-1.05	0.085

Other	996	1.23	0.99-1.54	0.061	**0.68**	**0.46-0.99**	**0.048**
						R^2 =0.020	*

*) Adjusted for variables statistically significant at Step 1: Sex, age, long education

**Step 3: Health behaviors and physical function**							
Smoking (ref: never smoked)	2024						
Current smoker	553	0.83	0.61-1.12	0.242	0.98	0.76-1.26	0.871
Former smoker	2686	0.99	0.83-1.18	0.924	0.88	0.59-1.33	0.549
Drinking (ref: no drinking)	1121						
less than before COVID-19	1023	0.87	0.69-1.12	0.291	0.93	0.65-1.33	0.689
unchanged	**2848**	**0.62**	**0.51-0.77**	**<0.001**	0.92	0.68-1.23	0.560
more than before COVID-19	271	0.91	0.62-1-.33	0.628	1.62	0.94-2.77	0.080
Diet (ref: unchanged junk food)	4305						
more junk food	472	1.49	1.15-1.94	0.003	1.17	0.76-1.79	0.483
more healthy eating	483	1.32	1.01-1.73	0.043	1.11	0.73-1.69	0.611
Activity (ref: only if necessary)	580						
light physical activity	3672	0.67	0.53-0.85	0.001	0.91	0.62-1.31	0.601
physical activity	1011	0.70	0.52-0.93	0.015	0.86	0.54-1.38	0.538
Physical Function	**5371**	**0.97**	**0.96-0.98**	**<0.001**	**0.95**	**0.94-0.97**	**<0.0001**
						R^2 =0.035	**

**) Adjusted for variables statistically significant at step 2: Sex, age, follow-up patients, treatment (ref: no treatment or hormone treatment)

**Step 4: Psychological and physical symtoms**							
Perceived stress	**5371**	**1.19**	**1.16-1.22**	**<0.001**	1.04	0.99-1.09	0.114
Depression	**5371**	**1.11**	**1.09.1.13**	**<0.001**	1.04	0.99-1.08	0.086
Anxiety	**5371**	**1.11**	**1.09-1.12**	**<0.001**	1.01	0.97-1.06	0.564
Fear of SARS-CoV-2 infection	**5371**	**1.05**	**1.04-1.07**	**<0.001**	0.99	0.97-1.01	0.385
Fear of cancer recurrence/progression	**5371**	**1.06**	**1.05-1.07**	**<0.001**	**1.02**	**1.00-1.04**	**0.012**
Tiredness	**5371**	**1.09**	**1.07-1.10**	**<0.001**	1.00	0.97-1.03	0.779
Pain	**5371**	**1.07**	**1.06-1.09**	**<0.001**	**1.03**	**1.01-1.05**	**0.009**
						R^2 =0.064	***

***) Adjusted for variables statistically significant at step 3: Sex, age, follow-up patients, physical function, treatment (ref: no treatment or hormone treatment)

**Step 5: Social support**							
Emotional	**5371**	**0.96**	**0.94-0.98**	**<0.001**	0.99	0.94-1.05	0.802
Informative	**5371**	**0.96**	**0.94-0.97**	**<0.001**	1.01	0.96-1.06	0.687
Instrumental	**5371**	**0.96**	**0.95-0.98**	**<0.001**	0.97	0.94.1.01	0.147
Social isolation	**5371**	**1.12**	**1.10-1.14**	**<0.001**	**1.06**	**1.03-1.10**	**<0.001**
						R^2 = 0.061	****

****) Adjusted for variables statistically significant at step 4: Sex, age, follow-up patients, pain, treatment (ref.: no treatment or hormone treatment), fear of cancer recurrence/progression
